# Cerebellar Atrophy Modulates Freezing of Gait in Multiple System Atrophy Through Cognitive Dysfunction

**DOI:** 10.1002/cns.71045

**Published:** 2026-07-27

**Authors:** Huaguang Yang, Liang Li, Zhi Wen, Lanhua Hu, Yunfei Zha

**Affiliations:** ^1^ Department of Radiology Renmin Hospital of Wuhan University Wuhan China

**Keywords:** cerebellum, cognition, freezing of gait, multiple system atrophy, neuroimaging

## Abstract

**Background:**

Cerebellar atrophy is a prevalent condition in multiple system atrophy (MSA) associated with cognitive impairment; however, whether this cognitive dysfunction contributes to freezing of gait (FOG) in affected patients remains unclear.

**Objective:**

To investigate whether cognitive deficits mediate the relationship between cerebellar atrophy and FOG in patients with MSA.

**Methods:**

Cognitive function was assessed in 106 patients with MSA: 58 with FOG (MSA‐FOG) and 48 without FOG (MSA‐nFOG) using standardized neuropsychological tests. Structural T1‐weighted MRI scans were processed using FreeSurfer and the Spatially Unbiased Infratentorial toolbox (SUIT) for cerebellar lobule segmentation. Multivariate linear regression and mediation analyses were conducted to examine associations among cerebellar lobular volumes, cognitive domains, and New Freezing of Gait Questionnaire (N‐FOGQ) scores.

**Results:**

Patients with MSA‐FOG exhibited deficits in executive function, visuospatial ability, attention, working memory, language, and delayed recall, alongside significant atrophy in global cerebellar gray matter and lobules I–X. Regression analyses revealed that Crus II atrophy independently predicted higher N‐FOGQ scores, alongside deficits in executive function and attention, with impairments in these two cognitive domains mediating the association between Crus II volume reduction and FOG severity.

**Conclusions:**

Reduced Crus II volume may be associated with severity of FOG through impaired executive function/attention; thus, cerebellar atrophy may serve as a potential biomarker for FOG in patients with MSA.

## Graphical abstract

Focusing on cerebellar pathology, this study identifies Crus II atrophy as a primary driver of FOG in multiple system atrophy. Mediation analysis reveals that attention and executive deficits partially mediate (38.57%/33.57%) the impact of Crus II volume loss on FOG severity. These findings establish a specific cerebellar‐cognitive‐motor axis, highlighting regional cerebellar integrity as a crucial neuroimaging biomarker for MSA.

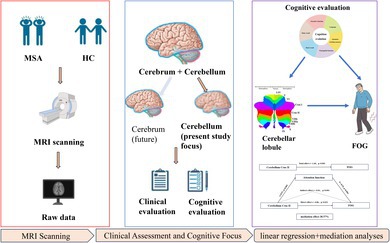

## 1 Introduction

Freezing of gait (FOG), defined as transient episodes of impaired forward progression during walking despite the intention to walk, is a prevalent and disabling symptom in multiple system atrophy (MSA). Importantly, FOG compromises mobility, increases fall risk [1, 2], and reduces quality of life [3, 4]. However, the underlying pathophysiology remains poorly characterized, and current treatments demonstrate limited efficacy. Accordingly, highlighting FOG constitutes a critical clinical challenge and a key focus for understanding the mechanisms involved in gait control.

Evidence from Parkinson's disease (PD) supports that there is a strong association between FOG and cognitive impairment, particularly deficits in executive dysfunction involving set‐shifting, attentional control, problem‐solving, and response inhibition [5, 6, 7]. Similarly, recent findings suggest that identical cognitive deficits may underlie FOG in MSA, though direct evidence for this relationship remains limited. FOG in MSA frequently manifests during motor transitions that require rapid switching (between motor programs, such as turning or navigating obstacles). This switching process relies heavily on the integration of competing motor, cognitive, and limbic inputs within basal ganglia‐cortical networks [8], which are severely disrupted in MSA. Importantly, complex gait tasks that demand heightened executive resources often precipitate FOG in MSA, further implicating attentional and inhibitory deficits in its pathophysiology. Visuospatial factors also appear to contribute, as FOG episodes can arise from maladaptive responses to action‐relevant visual cues, leading to disproportionate gait deterioration during spatial navigation tasks [9, 10]. Notably, the partial efficacy of laser‐cueing strategies in alleviating FOG extends to patients with MSA [11, 12], suggesting that both disorders may share common visuospatial regulatory mechanisms. Overall, although data on the direct relationship between cognition and FOG in MSA remain sparse, these cross‐disorder findings suggest that executive and visuospatial impairments are plausible contributors to FOG pathophysiology in MSA, warranting targeted investigation.

The cerebellum is a primary site of pathology in MSA, with neuroimaging studies consistently identifying cerebellar atrophy as a hallmark feature that correlates strongly with both motor and non‐motor symptom severity [13]. Beyond motor coordination, the cerebellum significantly contributes to cognitive processing (e.g., executive functions, language, attention behavioral regulation, and social cognition), via cortico‐cerebellar circuits [14]. Notably, cerebellar degeneration constitutes a significant risk factor for FOG in MSA [15], and lesions within midline cerebellar structures frequently induce a form of gait apraxia that phenotypically overlaps with FOG [16]. While a cerebellar‐ cognitive‐FOG axis has been documented in parkinsonian disorders, the specific pathophysiological mechanisms linking cerebellar structural integrity to cognitive impairment and subsequent FOG in MSA remain uncharacterized.

Although the association between cortical volumetric alterations and cognitive deficits in neurodegenerative syndromes is well established [13, 17], the cerebellar contribution to cognitive‐motor integration in MSA has remained underexplored. Thus, in this study, we investigated whether cognitive deficits underlie the link between cerebellar atrophy and FOG in patients with MSA. We hypothesized that cerebellar atrophy in MSA leads to executive and visuospatial impairments, which subsequently predispose patients to FOG. The associations between cerebellar volumetric indices and both FOG and cognitive severity were assessed using multivariate linear regression. Mediation analyses were subsequently conducted to determine how cognitive impairment connects cerebellar structural degeneration to FOG, thereby elucidating a potential mechanistic pathway specific to MSA.

## 2 Materials and Methods

### 2.1 Study Design and Participants

This cross‐sectional study was approved by the Ethics Committee of Renmin Hospital of Wuhan University (Approval No. WDRY2024‐K‐182) and was conducted in accordance with the Declaration of Helsinki (as revised in 2013). Written informed consent was obtained from all participants prior to enrollment. The study included 106 patients with MSA and 57 healthy controls (HCs). The MSA group comprised patients with at least a 12‐month history of a suspected movement disorder who were diagnosed with “probable” MSA by neurologists at Renmin Hospital of Wuhan University according to current clinical criteria [18]. The HC group consisted of individuals recruited through advertisements who had no evidence of brain lesions on conventional MRI and no history of other major neurological, psychiatric, or metabolic disorders (including diabetes and thyroid disease). The two cohorts were matched for age, sex, education level, and geographical location.

### 2.2 Clinical Assessments

FOG status was determined prospectively based on a dual‐source verification protocol: (1) direct observation of freezing episodes by movement disorder specialists during structured clinical assessments, and (2) documentation of community‐based episodes via standardized patient/caregiver diaries. Patients were classified into FOG (*n* = 58 “freezers” vs. *n* = 48 non‐freezers[nFOG]) was established per the Movement Disorder Society criteria using item 1 (“Do you experience episodes where your feet feel ‘glued’ to the floor?”) and item 3 (“Do you feel that your feet get glued to the floor while walking, turning, or attempting to initiate walking?”) of the New Freezing of Gait Questionnaire (N‐FOGQ) [19]. In cases of ambiguous self‐reports (e.g., due to limited patient insight into freezing episodes), clinical adjudication by neurologists determined FOG status through direct gait observation and video review. All patient responses underwent cross‐verification against caregiver reports and clinical records to ensure diagnostic accuracy. FOG severity was quantified using the validated N‐FOGQ score.

Clinicodemographic data were systematically collected by the attending neurologists on the day of the MRI through direct interviews. These variables included age, sex, education level, family medical history, disease duration, and the levodopa equivalent daily dose (LEDD), which was calculated using a standardized methodology [20], and included subcomponent quantification of levodopa preparations and dopamine receptor agonists.

Neuropsychological assessment encompassed comprehensive domain‐specific evaluations across multiple cognitive areas. Global cognition: Montreal Cognitive Assessment (MoCA); Verbal memory: Hopkins Verbal Learning Test (HVLT); Executive function: Trail‐Making Test Part B (TMT‐B; time‐to‐completion metric); Language: Boston Naming Test (BNT); Attention/working memory: Letter‐Number Sequencing (LNS); Visuospatial function: Judgment of Line Orientation (JLO). Motor and nonmotor symptom severity was quantified using: Motor function: Unified MSA Rating Scale (UMSARS) Part II and Hoehn & Yahr staging; Cerebellar ataxia: Scale for the Assessment and Rating of Ataxia (SARA); Depression: 24‐item Hamilton Depression Rating Scale (HAMD‐24).

### 2.3 MRI Protocol

Magnetic resonance imaging (MRI) was performed using a 3.0T scanner (GE Signa HDx, Milwaukee, WI, USA) equipped with a standard 16‐channel head coil. To minimize head motion and acoustic noise, restraining foam pads and earplugs were used. Prior to scanning, participants were instructed to remain still with their eyes closed, avoid falling asleep, and to refrain from directed or systematic thought. Subject wakefulness and compliance with these instructions were verified immediately following the scan session.

High‐resolution, three‐dimensional T1‐weighted structural images were acquired in the sagittal orientation using a magnetization‐prepared rapid acquisition gradient echo sequence. The acquisition parameters were defined as follows: matrix size, 256 × 256; slice total, 176; TR, 5000 ms; TE, 2960 ms; FOV, 256 × 256; flip angle, 12°, slice thickness = 1 mm without slice gap; distance factor, 0.5; and an isotropic voxel size 1.0 × 1.0 × 1.0 mm^3^.

### 2.4 Cerebellar Volumetry Analysis

Cerebellar volumetric quantification was performed using cerebellar gray matter (GM) and white matter (WM) probability maps generated by the Brain Extraction Tool (BET) and FMRIB's Automated Segmentation Tool (FAST) within FSL (v6.0). Cerebellar lobular segmentation was conducted using the Spatially Unbiased Infratentorial Toolbox (SUIT v6.5) integrated with Statistical Parametric Mapping v12 (SPM12, MATLAB R2023b). Briefly, this pipeline standardizes lobule dimensions to mitigate interindividual anatomical variability while preserving native‐space volumetric integrity. The processing sequence commenced with cerebellar isolation on high‐resolution 3DT1‐weighted structural images, followed by nonlinear registration of cropped T1 volumes to the SUIT template space. Concurrently, the GM and WM probability maps underwent identical nonlinear transformations using a 7° B‐spline interpolation to achieve precise lobular alignment within a standardized coordinate framework. Subsequently, an inverse nonlinear transformation remapped the SUIT and Buckner whole‐cerebellum atlas into each participant's native space, enabling accurate quantification of total cerebellar volume and lobule‐specific GM and WM compartments. The probability maps were thresholded to create binarized label maps using FSL, and all volumes were normalized to total intracranial volume (TIV) using a SIENAX‐derived scaling factor. Notably, vermal segmentation was excluded for participants in whom atlas‐based parcellation failed to yield reliable maps due to small structural size and high susceptibility to partial volume effects.

### 2.5 Cerebral Voxel‐Based Morphometry Analysis

Cerebral structural analysis was performed using voxel‐based morphometry (VBM8) within SPM12. Prior to preprocessing, all T1‐weighted images underwent quality control assessments to check for motion artifacts, intensity inhomogeneities, and adequate anatomical coverage. After manual reorientation to the anterior commissure–posterior (AC‐PC) commissure line, the images were segmented into GM, WM, and cerebrospinal fluid (CSF) using the “New Segment” algorithm with default tissue probability maps. Inter‐subject spatial normalization was executed via affine registration to the International Consortium for Brain Mapping (ICBM152) Montreal Neurological Institute (MNI) template, incorporating modulation to preserve the volumetric information of native‐space tissue segments. Spatial smoothing was subsequently applied using a 6 mm full‐width at half‐maximum (FWHM) isotropic Gaussian kernel (full‐width at half‐maximum = 6 mm), optimized to detect regional volume differences in neurodegenerative cohorts.

## 3 Statistical Analysis

Statistical analyses were performed using the R statistical software environment (version 3.1.3). The normality of data distribution was assessed using the Shapiro–Wilk test. Variables that exhibited substantial skewness were log‐transformed before further analysis while data that were non‐normally distributed were evaluated using non‐parametric equivalents. Continuous variables are presented as means ± standard deviation (SD) for normally distributed data, or as medians for non‐normally distributed data. Categorical variables are presented as frequencies and percentages.

Differences in cognitive domains between the MSA subgroups were evaluated using generalized linear models (GLMs) with age, sex, education level, and UMSARS Part II scores included as covariates; subgroup comparisons additionally included disease duration as a covariate. Cerebellar volume comparisons were performed using a multivariate GLM adjusted for TIV. The significance threshold was set at *p* < 0.05/*n* (Bonferroni‐corrected, where *n* = represents the number of demographic variables, cognitive domains, cerebral MRI metrics or cerebellar volumes evaluated within each analysis).

Associations between cerebellar lobular volumes and N‐FOGQ scores were evaluated using multiple linear regression. The models incorporated predictors in a stepwise manner: (1) demographic characteristics (age, sex and DD); (2) cerebral TIV and cerebellar metrics entered stepwise; (3) cognitive performance (with education added for cognitive models). Predictors were significant at *p* < 0.05 (Bonferroni‐corrected for multiple comparisons within each model).

Mediation analysis was conducted to determine whether cognitive impairment underlay the relationship between cerebellar atrophy and FOG severity. The three‐variable model specified cerebellar volume as the independent variable, FOG severity as the dependent variable, and cognitive performance as the mediator, with age, sex, education level, TIV, and UMSARS Part II scores included as covariates. Statistical significance was determined via bootstrapping with 5000 replications to calculate 95% confidence interval [CI]. A mediation effect was considered significant if the 95% CI excluded zero. The proportion = of the total effect mediated was calculated using the formula: (indirect effect/total effect) × 100%. These analyses were implemented using the PROCESS macro [21].

## 4 Results

### 4.1 Demographic Data

There were no significant differences in age, sex, education level, or HAMD‐24 scores between the MSA cohort and the HC group (all *p* > 0.05), indicating that the groups were well matched. Disease‐specific clinical variables, including disease duration, UMSARS scores, SARA scores, Hoehn & Yahr stage, and LEDD, are summarized in Table 1, alongside demographic profiles and N‐FOGQ scores for the clinical subgroups.

**TABLE 1:** Patient demographics and clinical characteristics.

Characteristics (Mean ± SD)	HC (*n* = 57)	MSA (*n* = 106)	MSA‐nFOG (*n* = 48)	MSA‐FOG (*n* = 58)	*p* ^a^	*p* ^b^
Age (years)	62.32 ± 5.14	63.78 ± 8.09	63.65 ± 8.64	63.90 ± 7.68	0.22	0.88
Gender (male: female)	24:33	51:55	27:21	24:34	0.46	0.13
Education	10.63 ± 2.23	9.89 ± 3.62	9.35 ± 3.23	10.33 ± 3.88	0.16	0.17
MSA‐P/MSA‐C	NA	46:60	23:25	23:35	NA	0.39
LEED	NA	491.01 ± 277.10	457.03 ± 225.96	519.12 ± 312.35	NA	0.24
Disease duration	NA	2.94 ± 1.26	2.88 ± 1.50	2.99 ± 1.04	NA	0.64
Hoehn and Yahr stage	NA	2.48 ± 0.77	2.43 ± 0.81	2.52 ± 0.74	NA	0.55
UMSARS score	NA	33.17 ± 15.18	30.23 ± 16.58	35.60 ± 13.58	NA	0.07
SARA score	NA	19.52 ± 6.53	18.43 ± 5.33	20.42 ± 7.30	NA	0.11
HAMD score	3.91 ± 2.39	4.20 ± 2.18	4.10 ± 2.33	4.28 ± 2.06	0.44	0.68
MOCA score	26.40 ± 1.56	22.86 ± 2.76	23.13 ± 2.81	22.64 ± 2.73	< 0.001	0.37
HVLT‐R: total recall score	22.11 ± 1.22	12.26 ± 5.37	13.21 ± 5.22	11.47 ± 5.42	< 0.001	0.09
HVLT‐R: short recall score	11.61 ± 0.49	8.44 ± 2.63	8.58 ± 2.47	8.33 ± 2.77	< 0.001	0.62
HVLT: delayed recall score	10.49 ± 1.00	3.83 ± 3.21	4.67 ± 3.15	3.14 ± 3.11	< 0.001	0.01
Boston naming test	28.86 ± 1.41	18.29 ± 7.57	20.17 ± 6.71	16.74 ± 7.94	< 0.001	0.02
Trail‐making, part B	127.33 ± 48.30	78.49 ± 10.66	113.31 ± 30.43	138.93 ± 56.84	< 0.001	0.01
Judgment of line orientation	14.00 ± 1.17	11.09 ± 3.44	12.00 ± 2.84	10.35 ± 3.73	< 0.001	0.01
Letter number sequencing	13.54 ± 1.21	10.04 ± 3.18	9.43 ± 3.20	10.78 ± 3.20	< 0.001	0.02

*Note:* *p* ^a^ values for the HC vs. MSA comparison; *p* ^b^ values for the MSA‐FOG vs. MSA‐nFOG comparison; *p* < 0.05 was considered statistically significant.

Abbreviations: HAMD‐24, 24 items Hamilton Depression Scale; HC, healthy controls; LEED, levodopa Equivalent dose; MOCA, Montreal Cognitive Assessment; MSA, multiple system atrophy; MSA‐FOG, multiple system atrophy with freezing of gait; MSA‐nFOG, multiple system atrophy without freezing of gait; P/C, Parkinson's type/Cerebellar type; SD, standard deviation; UMSARS, Unified Multiple System Atrophy Rating Scale.

### 4.2 Cognitive Performance

Compared with the HC group, the MSA cohort demonstrated significant impairments in global cognition and all assessed cognitive domains (executive function, visuospatial function, language, attention and working memory, and memory; all comparisons *p* < 0.05). Within the patient cohort, patients with MSA‐FOG exhibited greater cognitive impairment than those with MSA‐nFOG in executive function, attention and working memory, visuospatial function, and language. However, no significant differences were observed between these two clinical subgroups regarding immediate or delayed recall performance (Table 1).

### 4.3 Cerebral and Cerebellar Volumes Analysis

Between‐group differences in normalized cerebral and cerebellar volumes are outlined in Table 2. Compared with the HC group, the MSA cohort exhibited significantly lower normalized total intracranial volume (TIV), cerebral GM and WM volumes, alongside widespread reductions in total cerebellar volume that spanned both the anterior and posterior compartments and lobules I–X (all *p* < 0.05; covariates: adjusted for age, sex, and disease duration). Compared with the MSA‐nFOG subgroup, the MSA‐FOG subgroup demonstrated significantly greater volume reductions in normalized cerebral GM, total cerebellar volume, and across all individual lobules from I to X (all *p* < 0.05; covariates: age, sex, and disease duration). In contrast, normalized TIV and cerebral WM volumes did not differ significantly between the two patient subgroups.

**TABLE 2:** MRI metrics for all subjects included in the analysis.

	HC (*n* = 57)	MSA (*n* = 106)	MSA‐nFOG (*n* = 48)	MSA‐FOG (*n* = 58)	*p* ^a^	*p* ^b^
Cerebral TIV	1437.20 ± 110.20	1369.44 ± 121.26	1390.70 ± 109.10	1351.85 ± 128.74	**0.001**	0.096
Cerebral GM volume	619.06 ± 40.89	559.75 ± 50.71	575.15 ± 49.29	547.00 ± 48.66	**< 0.001**	**0.004**
Cerebral WM volume	553.53 ± 51.35	526.97 ± 60.40	534.80 ± 56.99	520.49 ± 62.83	**0.005**	0.222
Global cerebellar volume	90.69 ± 9.05	79.33 ± 12.94	83.31 ± 13.48	76.04 ± 11.58	**< 0.001**	**0.004**
Anterior cerebellar volume	12.17 ± 1.12	10.71 ± 1.79	11.16 ± 1.85	10.34 ± 1.66	**< 0.001**	0.017
Posterior cerebellar volume	78.52 ± 8.02	68.62 ± 11.23	72.15 ± 11.69	65.70 ± 10.03	**< 0.001**	**0.003**
Lobule I‐IV	5.08 ± 0.46	4.39 ± 0.78	4.59 ± 0.79	4.22 ± 0.72	**< 0.001**	0.012
Lobule V	7.09 ± 0.69	6.33 ± 1.03	6.57 ± 1.08	6.12 ± 0.96	**< 0.001**	0.026
Lobule VI volume	16.35 ± 1.64	14.61 ± 2.34	15.20 ± 2.38	14.12 ± 2.22	**< 0.001**	0.017
Crus I volume	19.62 ± 2.18	17.25 ± 2.95	18.31 ± 3.11	16.53 ± 2.61	**< 0.001**	**0.005**
Crus II volume	19.62 ± 2.18	12.31 ± 1.89	18.13 ± 3.11	11.79 ± 1.62	**< 0.001**	**0.001**
Lobule VIIb volume	7.69 ± 0.88	6.65 ± 1.14	7.02 ± 1.21	6.35 ± 0.99	**< 0.001**	**0.002**
Lobule VIIIa volume	8.19 ± 0.86	7.16 ± 1.22	7.54 ± 1.28	6.84 ± 1.07	**< 0.001**	**0.003**
Lobule VIIIb volume	6.15 ± 0.60	5.35 ± 0.96	5.65 ± 1.00	5.11 ± 0.86	**< 0.001**	**0.004**
Lobule IX	5.48 ± 0.58	4.57 ± 0.97	4.87 ± 0.92	4.31 ± 0.94	**< 0.001**	**0.002**
Lobule X	0.92 ± 0.11	0.73 ± 0.19	0.78 ± 0.19	0.69 ± 0.18	**< 0.001**	0.007

*Note:* Volumes are expressed as mean ± SD deviation. *p* ^a^ Values for the HC vs. MSA comparison. *p* ^b^ Values for the MSA‐FOG vs. MSA‐nFOG comparison. Statistical significance was set at *p* < 0.05. For multiple comparisons of the 10 cerebellar lobules, the significance level was adjusted to *p* < 0.005 using Bonferroni correction. Statistically significant differences are indicated in bold.

Abbreviations: HC, healthy controls; MSA, multiple system atrophy; MSA‐FOG, multiple system atrophy with freezing of gait; MSA‐nFOG, multiple system atrophy without freezing of gait; MSA‐P/MSA‐C, Parkinson's type/Cerebellar type MSA; TIV, total intracranial volume.

### 4.4 Association Among Cerebellum Atrophy, Cognitive Impairment Profiles, and FOG Severity in MSA‐FOG Group (Cerebellum‐FOG and Cerebellum‐Cognitive)

**FIGURE 1:**
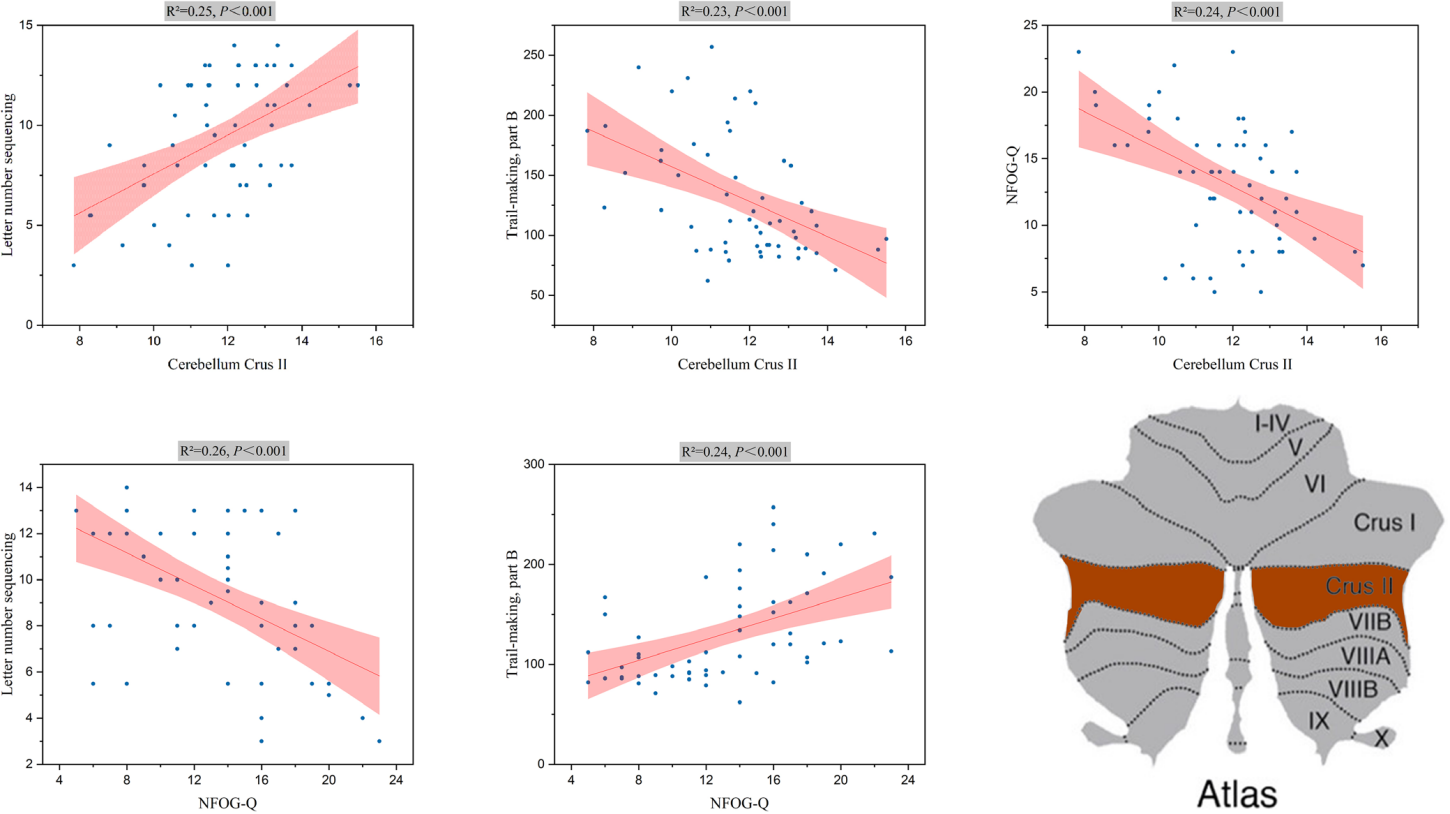
Cross‐sectional association among the cerebellum Crus II volume, N‐FOGQ score and cognitive scores in MSA‐FOG group. Scatter plots show a significantly positive correlation between Crus II GM volume and attention, between N‐FOGQ score and executive function, but reverse correlation between cerebellum Crus II volume and executive function/N‐FOGQ score and between N‐FOGQ score and attention function for multiple linear regression after controlling for age, sex, education, UMSARS, LEED and cerebral volume.

Multivariate linear regression analysis showed that a lower mean Crus II volume was significantly associated with a higher TMT‐B score (β = −0.419, *p* < 0.001), lower LNS score (*β* = 0.452, *p* < 0.001) in the MSA‐FOG group, after adjusting for multiple covariates (clinical variables: age, sex, UMSARS and LEED. Cerebral variables: Cerebral TIV, Cerebral GM and Cerebral WM). Furthermore, a lower cerebellar Crus II volume was associated with higher N‐FOGQ scores (*β* = −0.470, *p* < 0.001), after adjusting for these covariates (Figure 1).

In the MSA‐FOG subgroup, executive function (TMT‐B: *β* = 0.032, *p* = 0.013) and attention/working memory (LNS: *β* = −0.486, *p* = 0.015) showed significant associations with FOG severity. In contrast, no significant associations between cognitive performance and cerebellar atrophy were observed in the MSA‐nFOG subgroup, after adjusting for relevant covariates in the regression analysis. The detailed results are presented in Table 3.

**TABLE 3:** Results of the hierarchical multiple linear regression analysis when considering cerebellar lobule volumes in MSA‐FOG patients' group.

	Model				Independent predictors		
	*R* ^2^	*R* ^2^ change	*F*	*p*	*β*	*T*‐value	*p*
**Executive functions**	0.399	0.366	14.86	< 0.001			
**Gender**					0.316	2.778	0.008
Education					−0.176	−1.504	0.138
**Crus II**					−0.419	−3.855	< 0.001
**NFOGQ**	0.302	0.277	17.362	< 0.001			
**Crus II**					−0.470	−4.167	< 0.001
**Attention and working memory**	0.282	0.256	14.785	< 0.001			
Duration					0.190	1.614	0.112
**Crus II**					0.452	3.845	< 0.001

*Note:* Bonferroni correction was applied for multiple comparisons; statistically significant independent variables are indicated in bold. “Duration” refers to disease duration (years).

### 4.5 Cognitive Function Impairment Underlies the Association Between Cerebellar Atrophy and FOG Symptoms

Executive function, attention, and working memory significantly mediated the association between cerebellar Crus II volume and N‐FOGQ scores after adjusting for covariates (i.e., age, sex, education level and UMSARS).

**FIGURE 2:**
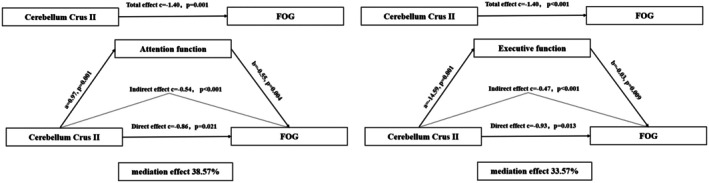
Mediation analysis. Executive/attention had a significant indirect effect (𝛽 = −0.54, 𝛽 = −0.47), mediating (33.57%, 38.57%) effect for the relationship between cerebellum crus II and FOG severity. Mediation analysis was performed while controlling for age, sex, education, UMSARS, LEED and cerebral volume.

The mediation effect of executive function on FOG symptoms was significant, with a proportion mediated (PM = 33.57%; total effect *c* = −1.40, *p* < 0.001; direct effect *c* = −0.93, *p* = 0.013; indirect effect *β* = −0.47; [95% CI] = −0.86 to −0.16). Similarly, the mediation effect of attention and working memory (PM = 38.57%; total effect *c* = −1.40, *p* = 0.001; direct effect *c* = −0.86, *p* = 0.021; indirect effect *β* = −0.54; [95% CI] = −0.98 to −0.11) on the FOG symptoms caused by cerebellum atrophy (Crus II) was significant (Figure 2).

## 5 Discussion

FOG causes postural instability and gait disturbances, frequently leading to falls that severely diminish patient quality of life. Consequently, elucidating the underlying pathophysiological mechanisms is crucial. This study focused on the cerebellum, a structure with well‐established vulnerability in MSA. Two key findings emerged regarding FOG in patients with MSA. Firstly, cerebellar atrophy, particularly within Crus II, was significantly associated with FOG severity, demonstrating a distinct structural correlation. Secondly, deficits in executive function and attention/working memory partially mediated the relationship between cerebellar volume reduction and FOG symptoms. This underscores the pivotal role of cognitive‐motor interactions in the pathophysiology of gait impairment. Collectively, these results indicate that cerebellar atrophy may serve as a potential biomarker for FOG in MSA. Furthermore, the findings suggest that integrated therapeutic strategies targeting both the motor and cognitive domains may improve patient outcomes and quality of life.

Despite the comparable clinical severity between the MSA‐FOG and MSA‐nFOG subgroups, the MSA‐FOG cohort demonstrated significantly poorer performance in specific cognitive domains, particularly executive function and attention, even after controlling for UMSARS Part II scores. This indicates that an independent association exists between FOG and deficits in distinct cognitive domains in MSA, suggesting that specific impairments may interact with and exacerbate freezing episodes. Whether motor symptom severity correlates directly with both FOG and cognition remains an important consideration in the field. Although overall disease progression is typically associated with an increased prevalence of FOG and cognitive decline [22, 23], emerging evidence has revealed a more complex relationship. Clinical data indicate that these features are driven by distinct pathological processes. Stewart et al. proposed that FOG arises from a dysfunctional feedback loop involving the norepinephrine, cholinergic, and dopaminergic systems, with extranigral neurotransmitter circuits operating independently of primary dopaminergic pathology [24]. Non‐dopaminergic mechanisms, including norepinephrine depletion and neuroinflammation, may drive FOG progression independently of general motor symptom decline. This anatomical and chemical distinction is supported by multicenter evidence showing that while cognitive and motor functions decline concurrently in PD, progressive supranuclear palsy, and corticobasal syndrome, such a correlation is notably absent in MSA [25]. The lack of clear association among cognitive impairment, motor symptom severity, and FOG in MSA suggests that disease‐specific pathophysiological mechanisms are at play. Because cross‐sectional analyses capture only transient disease states, longitudinal studies tracking the transition from non‐freezing to freezing states—while monitoring cognitive trajectories and non‐dopaminergic biomarkers—are essential for clarifying these underlying pathways.

The pathophysiology of FOG is multifactorial, involving dysfunctional multisystem feedback loops, non‐dopaminergic pathways, motor circuit abnormalities, and cognitive‐motor integration deficits. Within the MSA‐FOG cohort, cerebellar atrophy was significantly associated with both domain‐specific cognitive impairment and FOG severity. Furthermore, a strong negative correlation was observed between executive/attentional function and N‐FOGQ scores. Mediation analysis revealed that executive function and attention/working memory partially mediated the relationship between cerebellar Crus II volume and FOG severity (with mediation effect: 33.57% and 38.57%, respectively) after adjusting for demographic variables and cerebral volumes. These findings align with the cognitive gait interference hypothesis. Firstly, this partial mediation suggests that a dual mechanism accounts for these findings: an indirect “cognitive‐motor” route and a significant direct “structural‐motor” pathway. The indirect route (represented by the ~37% average mediation effect) indicates that Crus II atrophy contributes to FOG by disrupting the cognitive modulation of movement, particularly in tasks requiring high executive load and attentional shifting. However, the presence of a robust direct effect (*p* < 0.05) implies that cerebellar atrophy also influences FOG through mechanisms independent of the cognitive domains assessed. This direct effect likely reflects the intrinsic role of the cerebellum in basic motor execution, such as the regulation of gait cadence, stride‐to‐stride variability, and the integration of multi‐sensory feedback required for postural stability. By distinguishing between these direct and indirect contributions, these findings clarify how cerebellar degeneration drives the complex FOG phenotype in MSA. Secondly, this involvement of Crus II aligns with recent functional neuroimaging evidence in MSA. Specifically, research on topological organization indicates that MSA patients with FOG exhibit extensive disruption in both low‐ and high‐order functional networks, with significant alterations in nodal centralities within the cerebellar network, including Crus II [26]. These findings highlight the importance of Crus II as a critical node within the cognitive‐motor integration network. In PD, FOG severity is closely linked to cognitive impairment; however, cerebellar structural alterations are often inconsistent, and cerebellar atrophy does not act as a primary mediator of these cognitive‐motor interactions [27, 28, 29]. In contrast, MSA is characterized by severe olivopontocerebellar degeneration, resulting in a distinct, primary cerebellar‐cognitive‐FOG coupling. Notably, while cerebellar structural atrophy correlated strongly with FOG severity in this cohort, functional studies have yielded mixed results. For instance, while abnormal functional connectivity between the thalamus and cerebellum has been observed in MSA‐FOG, some resting‐state fMRI analyses found no direct correlation between cerebellar functional activity (such as degree centrality) and FOG scores [30]. This discrepancy between structural and functional findings suggests that while functional alterations may reflect state‐dependent changes or compensatory mechanisms, structural atrophy in Crus II may represent a more stable and direct biomarker for the progression of FOG in MSA. Given the cerebellum's central role of the cerebellum in MSA pathology [17, 31], our findings establish: Firstly, the current data demonstrate that cerebellar atrophy is significantly associated with executive and attentional impairment in MSA. Secondly, Crus II subregions modulate FOG severity. Thirdly, this specific cognitive impairment partially mediates the relationship between cerebellar atrophy and the FOG pathway. This points to an MSA‐specific cascade from Crus II atrophy to executive and attentional dysfunction in FOG. Crucially, the dissociation from PD mechanisms—where cerebellar atrophy associates with FOG without mediating cognitive pathways—highlights distinct disease‐specific architectures. To our knowledge, this study provides the first evidence of subregional cerebellar contributions to FOG via discrete cognitive mediators, advancing the paradigm of cerebellar‐cognitive‐motor integration in MSA.

In addition to executive and attentional deficits, previous studies in PD have demonstrated that cerebellar atrophy is correlates with memory and language impairments [32, 33, 34]. Notably, the MSA‐FOG patients in our cohort showed more attentional and memory deficits than non‐FOG counterparts, without significant visuospatial differences. The role of visuospatial dysfunction in FOG remains controversial: some studies report significant visuospatial deficits, while others highlight predominant executive/attentional impairments [33]. These discrepancies likely reflect differences in disease etiology (e.g., PD vs. MSA) or assessment tools. Furthermore, the sensitivity of cognitive batteries to detect FOG‐related deficits varies across studies, as certain domains (e.g., visuospatial function) may not be adequately captured by standard tests. This variation reveals a fundamental methodological limitation in FOG research: because cognitive correlates depend heavily on the testing instruments, these tools must be rigorously validated, and cautious interpretation of domain‐specific findings must be interpreted with caution.

## 6 Limitation

This study has three principal limitations. Firstly, the cross‐sectional design precludes establishing the temporal dynamics of cerebellar atrophy or causal relationships among cerebellar structural changes, cognitive decline, and FOG progression in MSA. Secondly, although structural volumetric alterations were quantified, functional reorganization within the cerebellar‐cortical networks was not analyzed. This omission potentially obscured compensatory mechanisms relevant to cognitive adaptation [35]. Thirdly, although the MSA‐P (parkinsonian) and MSA‐C (cerebellar) subtypes were comparably distributed across groups, these classifications rely primarily on clinical motor phenomenology. Significant clinical heterogeneity persists within each subtype, particularly in the severity and distribution of autonomic dysfunction [36]. Because this study focused specifically on FOG as a core symptom of MSA, independent of conventional motor‐based subtype categorization, future research should investigate the differential relationships between MSA motor subtypes (MSA‐P vs. MSA‐C) and FOG phenotypes to clarify subtype‐specific mechanisms and optimize targeted interventions.

## 7 Conclusion

Cerebellar atrophy, specifically in Crus II, is independently associated with FOG severity in MSA. Executive function and attention/working memory deficits partially mediate this relationship, establishing a cerebellar‐cognitive axis in FOG pathophysiology. These findings indicate that cerebellar structural integrity is a key mechanistic contributor to gait disturbances in MSA. Consequently, regional cerebellar atrophy may serve as a structural biomarker for FOG in this population.

## Author Contributions

Huaguang Yang and Yunfei Zha conceived the study. Huaguang Yang, Zhi Wen, Lanhua Hu, and Liang Li collected and analyzed the data. Zhi Wen contributed to writing the review and language polish. Huaguang Yang writing the draft, Yunfei Zha revised the article, and there was final approval of the version to be submitted. All authors read and approved the final manuscript.

## Funding

This study was supported by grants from the National Natural Science Foundation of China for its funding of this experiment [grant numbers 82302143].

## Ethics Statement

This study was approved by the Ethics Committee of the Wuhan University Renmin Hospital Ethics Committee (WDRY2024‐K‐182). All subjects signed a written informed consent form and all methods used in the present study were performed in accordance with the Declaration of Helsinki.

## Consent

The authors have nothing to report.

## Conflicts of Interest

The authors declare no conflicts of interest.

## Data Availability

The datasets used and/or analyzed during the current study are available from the corresponding author on reasonable request.
